# A Triple-Arginine Motif in the Amino-Terminal Domain and Oligomerization Are Required for HIV-1 Inhibition by Human MX2

**DOI:** 10.1128/JVI.00169-15

**Published:** 2015-02-11

**Authors:** Caroline Goujon, Rebecca A. Greenbury, Stelios Papaioannou, Tomas Doyle, Michael H. Malim

**Affiliations:** Department of Infectious Diseases, King's College London, London, United Kingdom

## Abstract

We have employed molecular genetic approaches to understand the domain organization of the HIV-1 resistance factor myxovirus resistance 2 (MX2). First, we describe an essential triple-arginine motif in the amino-terminal domain. Second, we demonstrate that this 91-residue domain mediates antiviral activity when appended to heterologous proteins, and we provide genetic evidence that protein oligomerization is required for MX2 function. These insights will facilitate future work aiming to elucidate MX2's mechanism of action.

## TEXT

Type I interferons (IFNs) are produced in response to acute virus infection and evoke an antiviral state in susceptible cells through the expression of a diverse array of IFN-stimulated genes (ISGs) (1–3). Prominent among these are the dynamin-like guanosine triphosphatases (GTPases) *MX1* (or *MXA*) and *MX2* (or *MXB*) (4–6). The broad antiviral properties of MX1 have been recognized for many years, with the inhibitory effects on the orthomyxoviruses influenza A virus and Thogoto virus having been defined in detail (4, 5). In contrast, MX2, which previously had not been ascribed an antiviral function, was recently found to be a suppressor of primate immunodeficiency viruses, and particularly human immunodeficiency virus type 1 (HIV-1), but not of other retroviruses, such as murine leukemia virus (MLV) (7–9).

MX1 and MX2 each comprise an amino-terminal GTPase domain and a carboxy-terminal stalk domain that are connected by a tripartite bundle signaling element (BSE). A disordered loop (L4) in the stalk domain of MX1 interacts with orthomyxovirus nucleoproteins, and this is thought to promote the assembly of higher-order MX1 oligomers, GTPase activation and GTP hydrolysis, and conformational changes that disrupt viral replication complexes and their function (5, 10–12). MX2 antiviral function appears to be quite different: GTPase activity is dispensable (7, 9, 13), oligomerization beyond dimers appears unnecessary for viral suppression (14, 15), and antiviral specificity is determined by its amino-terminal 91 residues rather than L4 (13, 16, 17). Current models suggest that human MX2 recognizes the capsid (CA) lattice of HIV-1 reverse transcription complexes (RTCs) (7–9, 14, 17) and that inhibition occurs at a late postentry stage that is reflected as suppressed nuclear import and proviral formation (7, 9, 18).

**FIG 1:**
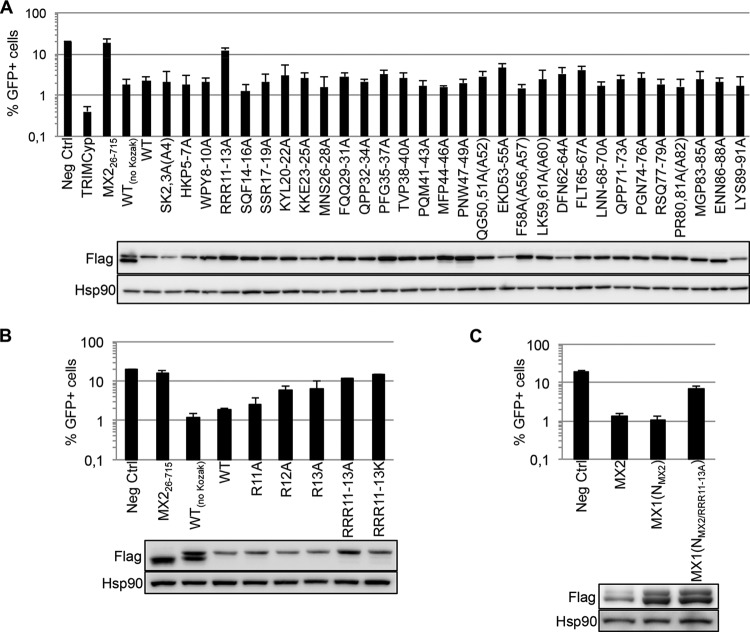
Definition of an essential triple-arginine motif in the amino-terminal domain of human MX2. (A) Alanine scanning mutagenesis of the amino-terminal domain of MX2. (Upper panel) U87-MG/CD4/CXCR4 cells were transduced with EasiLV expressing CD8 (Neg Ctrl), TRIMCyp, MX2 short isoform (MX2_26–715_), wild-type (WT) MX2 without a Kozak sequence [MX2_(no Kozak)_], or a series of WT and alanine scanning mutants (as indicated) bearing the Kozak sequence. The cells were treated with doxycycline (0.5 μg/ml) for 72 h and challenged with an HIV-1-based lentiviral vector expressing GFP (HIV-1/GFP) at a multiplicity of infection of 0.2. The percentage of GFP-expressing cells was evaluated by flow cytometry 2 days later. Mean percentages of transduced cells from three independent experiments are shown. (Lower panel) Immunoblot analysis of parallel samples from upper panel. Protein levels of Flag-tagged MX2 proteins were determined, and Hsp90 served as a loading control. (B) Effect of individual-alanine and triple-lysine mutations in the triple-arginine motif. U87-MG/CD4/CXCR4 cells expressing CD8 (Neg Ctrl), MX2_26–715_, WT_(no Kozak)_ or Kozak sequence-bearing WT MX2, or single-alanine or triple-lysine MX2 mutations were challenged with HIV-1/GFP vector at a multiplicity of infection of 0.2, and infection was analyzed by flow cytometry 2 days later, as in panel A. Mean percentages of transduced cells from three independent experiments are shown. (Lower panel) Immunoblot analysis of parallel samples from upper panel was performed as for panel A. (C) Effect of disrupting the triple-arginine motif in the context of MX1(N_MX2_) chimera. U87-MG/CD4/CXCR4 cells expressing CD8 (Neg Ctrl), MX2, WT MX1(N_MX2_), and the RRR11–13A mutant were challenged with HIV-1/GFP at a multiplicity of infection of 0.2. Mean percentages of transduced cells from four independent experiments are shown. (Lower panel) Immunoblot analysis of parallel samples from upper panel was performed as for panel A.

As a step toward understanding the molecular basis for MX2 function, we sought to define the regions and residues of the amino-terminal 91 amino acids required for activity. Accordingly, scanning triple-alanine substitution mutations were introduced across the entire region in the context of the tetracycline-inducible lentiviral vector construct pEasiLV-MX2_Kozak_, which expresses a carboxy-terminally Flag-tagged version of the full-length (715-amino-acid) human protein (13). Approximately 1 × 10^5^ to 2 × 10^5^ U87-MG/CD4/CXCR4 cells were transduced with 293T-derived high-titer EasiLV stocks encoding these mutants, as well as corresponding wild-type, negative-control (CD8 or the short isoform of MX2, MX2_26–715_), or positive-control (TRIMCyp) stocks (7). Following tetracycline induction, the cultures were challenged with vesicular stomatitis virus G protein (VSV G)-pseudotyped green fluorescent protein (GFP)-expressing stocks of an HIV-1-based lentiviral vector (HIV-1/GFP) (19), and infection efficiency was enumerated in EasiLV-transduced cell populations by flow cytometry at 48 h as the percentage of GFP-positive cells (7) (Fig. 1A). As previously established, MX2 suppresses HIV-1 infection by at least 90% (7, 9). Out of these 30 mutant proteins, only one, where the three arginines at positions 11 to 13 had been replaced (RRR11–13A), was inactive; indeed, the remaining mutants displayed minimal variation from full suppressive function. Immunoblotting for the Flag tag confirmed that all MX2 proteins were expressed, though EKD53–55A accumulated slightly less well, likely accounting for its apparent marginal reduction in potency.

We next replaced arginines 11 to 13 individually with alanine (Fig. 1B). Although each residue appears to contribute to the full antiviral behavior of MX2, the arginines at positions 12 and 13 are more influential. We also assessed the importance of charge at these positions by replacing all three arginines with lysines. As with the triple-alanine substitution, this protein was nonfunctional, indicating that antiviral activity requires the presence of arginines at these positions rather than positively charged amino acids. Previous work has established that the transfer of the amino-terminal 91 amino acids of MX2 to MX1 confers a robust HIV-1-inhibitory phenotype (13). Consistent with the above observations, introduction of the RRR11–13A mutation into the MX1(N_MX2_) chimera also abrogated HIV-1 inhibition (Fig. 1C). Taken together, we conclude that the triple-arginine motif at positions 11 to 13 of human MX2 is essential for anti-HIV-1 function.

**FIG 2:**
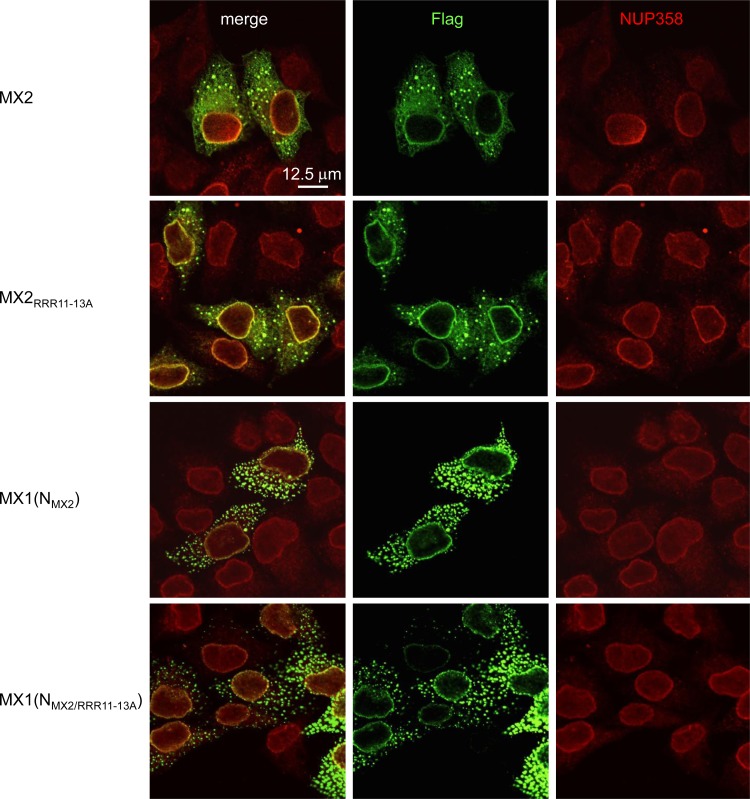
Indirect immunofluorescence analysis of wild-type and RRR11–13A versions of MX2 and MX1(N_MX2_). HeLa cells were seeded on glass coverslips and transfected with pCAGGS-based vectors expressing Flag-tagged wild-type or RRR11–13A forms of MX2 or MX1(N_MX2_) and fixed 16 h posttransfection. MX proteins and the NE were visualized using Flag- or NUP358-specific antibodies, respectively, and confocal microscopy (13). Bar, 12.5 μm.

Indirect immunofluorescence analysis of transiently transfected HeLa cells revealed that both wild-type MX2 and the RRR11–13A mutant accumulated at the nuclear envelope (NE), throughout the cytoplasm, and in large cytoplasmic bodies/granules (Fig. 2). Similarly, both the wild-type MX1(N_MX2_) chimera and its RRR11–13A derivative also displayed similar patterns of localization. These observations indicate that modified subcellular localization does not account for the lack of antiviral function of RRR11–13A mutant MX proteins.

**FIG 3:**
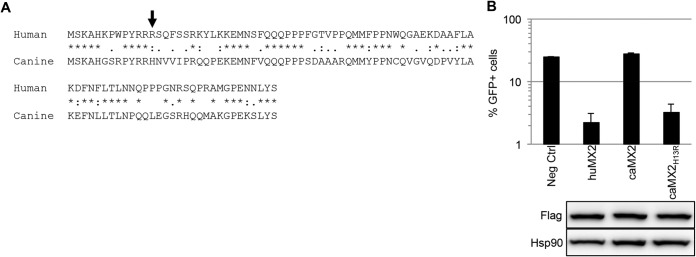
A histidine-to-arginine alteration at position 13 in canine MX2 confers potent anti-HIV-1 function. (A) ClustalW sequence alignment of the first 91 amino acids of human and canine MX2. Asterisks, identical amino acids; colons, conserved substitutions; periods, semiconserved substitutions. (B) U87-MG/CD4/CXCR4 cells were transduced with EasiLV expressing CD8 (Neg Ctrl) or amino-terminally Flag-tagged human MX2 (huMX2), canine MX2 (caMX2), or canine MX2_H13R_. The cells were treated with doxycycline for 72 h and challenged with HIV-1/GFP at a multiplicity of infection of 0.25. The percentage of GFP-expressing cells was evaluated by flow cytometry 2 days later. Mean percentages of transduced cells from three independent experiments are shown. (Lower panel) Immunoblot analysis of parallel samples from the upper panel was performed as for Fig. 1A. Of note, the amino-terminal Flag prevents leaky scanning, resulting in exclusive production of the long isoform of MX2.

The canine *MX2* ortholog does not inhibit HIV-1, but replacement of the amino-terminal 29 amino acids of the canine protein with those from the human counterpart creates a chimeric protein as effective as human MX2 in suppressing HIV-1 infection (16). Inspection of this region reveals 15 amino acid differences (Fig. 3A), with the critical arginine at position 13 being histidine in the canine protein (indicated with an arrow). As expected, canine MX2 does not exhibit an anti-HIV-1 phenotype in our system, but we found that replacing this single histidine with arginine, caMX2_H13R_, confers inhibitory function to a level close to that of human MX2 (Fig. 3B). This finding further underscores the critical contribution of the triple-arginine motif to the anti-HIV-1 properties of MX2.

**FIG 4:**
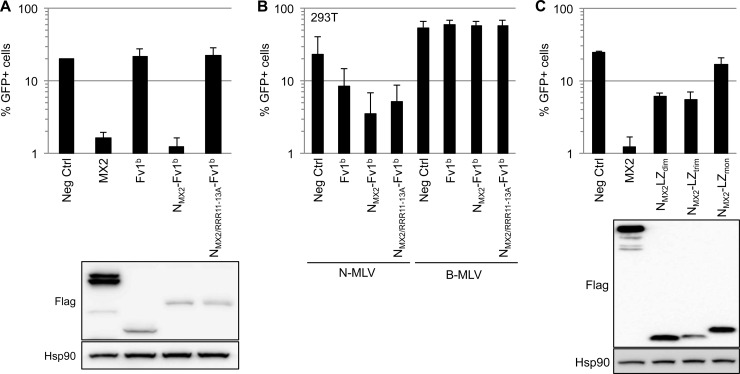
The amino-terminal domain of MX2 inhibits HIV-1 infection when transferred to heterologous scaffolds. (A) Fusion of the amino-terminal domain of MX2 to the amino terminus of Fv1^b^. U87-MG/CD4/CXCR4 cells were transduced with EasiLV expressing CD8 (Neg Ctrl), MX2, or amino-terminally Flag-tagged Fv1^b^, N_MX2_-Fv1^b^, or N_MX2/RRR11–13A_-Fv1^b^; treated with doxycycline for 3 days; and challenged with HIV-1/GFP. The percentage of infected cells was analyzed 2 days later by flow cytometry. Mean percentages of transduced cells from four independent experiments are shown. (Lower panel) Immunoblot analysis of parallel samples from the upper panel was performed as for Fig. 1A. (B) The N_MX2_-Fv1^b^ fusions inhibit infection by N-MLV. 293T cells were transduced with EasiLV expressing CD8 (Neg Ctrl), Fv1^b^, N_MX2_-Fv1^b^, or N_MX2/RRR11–13A_-Fv1^b^ and treated with doxycycline (2.5 μg/ml) for 3 days. The cells were then challenged with similar volumes (4 μl) of GFP-encoding N-MLV and B-MLV viral stocks (produced by 293T cotransfection of pMD.G, p13077, and pCIG3N or pCIG3B expression plasmids, respectively) (21, 27), and infection efficiency was analyzed by flow cytometry 2 days later. Mean percentages of transduced cells from three independent experiments are shown. (C) Dimeric and trimeric leucine zipper fusion proteins containing the amino-terminal domain of MX2. U87-MG/CD4/CXCR4 cells were transduced with EasiLV expressing CD8 (Neg Ctrl), MX2, and carboxy-terminally Flag-tagged fusion proteins containing the amino-terminal domain of MX2 grafted to different versions of the GCN4 leucine zipper, forming either dimers, trimers, or monomers (N_MX2_-LZ_dim_, N_MX2_-LZ_trim_, and N_MX2_-LZ_mon_, respectively); treated with doxycycline for 3 days; and challenged with HIV-1/GFP. The percentage of infected cells was analyzed 2 days later by flow cytometry. Mean percentages of transduced cells from three independent experiments are shown. (Lower panel) Immunoblot analysis of parallel samples from the upper panel was performed as for Fig. 1A.

Given that transfer of the amino-terminal 91 amino acids of MX2 onto MX1 bestows full anti-HIV-1 function (13), we wished to determine whether this domain would still have this capability when appended to an entirely unrelated protein. We initially chose to use mouse Fv1^b^ as the substrate since this protein is an inhibitor of retrovirus infection (N-tropic NLV but not HIV-1) (20, 21), suppression is manifested as a lack of viral cDNA integration (22, 23), and it is naturally oligomeric (24). Remarkably, fusing residues 1 to 91 of MX2 to the amino terminus of Fv1^b^ (N_MX2_-Fv1^b^) conferred potent HIV-1-inhibitory activity, whereas the parental Fv1^b^ protein had no effect (Fig. 4A). Importantly, a genetic constraint of MX2 function was faithfully preserved, as introduction of the RRR11–13A mutation into this chimeric protein abrogated activity. As a confirmation of Fv1^b^ functionality, both fusion proteins suppressed N-MLV (but not B-tropic MLV) infection as efficiently as the wild-type protein (Fig. 4B). Therefore, the amino-terminal domain of human MX2 is the only element of MX1/MX2 that is necessary for HIV-1 inhibition in the context of a heterologous fusion partner.

In a final series of experiments, we turned to a system where the oligomeric status of the MX2 fusion partner can be experimentally manipulated: namely, the well-characterized leucine zipper domain of yeast GCN4 (25). We therefore placed residues 1 to 91 of MX2 at the amino terminus of monomeric, dimeric, and trimeric versions of this leucine zipper (25, 26). As shown in Fig. 4C, the fusions containing the dimeric or trimeric zippers each suppressed HIV-1 by ∼80%, whereas the monomeric protein had no effect. In sum, the amino terminus of MX2 is sufficient to inhibit HIV-1 infection, provided that it is contained within a protein that can oligomerize, at least into dimers.

Here, we have employed site-directed mutagenesis to define an essential triple-arginine motif within the amino-terminal domain of human MX2 (Fig. 1 and 3), findings that are consistent with analyses of deletion mutants (17, 18). Previous work has revealed that residue 37 can also play a role in suppression mediated by the MX2 protein of macaque (16), but our alanine scanning failed to register the importance of this amino acid. Importantly, the triple-arginine motif is required for HIV-1 suppression irrespective of the scaffold to which the amino-terminal domain is attached, indicative of a vital role in MX2 function (Fig. 1 and 4).

A central question for unraveling the molecular basis for MX2 action is, therefore, identification of the relevant interacting partners for this element. The most likely possibilities are the RTC CA lattice and/or cellular factors. Although interactions between MX2 and viral cores or *in vitro*-assembled CA-containing nanotubes have been reported, their relevance for the inhibition of infection is currently uncertain, as they do not conform to the genetic determinants of HIV-1 suppression in that MX2-resistant CA proteins still interact with MX2 (14, 17). Future work will also explore potential interactions with host factors. Given that MX2's amino-terminal domain is sufficient to confer NE localization (13) and nuclear localization signals are frequently rich in lysines and/or arginines, the finding that the RRR11–13A protein displays the same staining pattern as that of the wild-type protein (Fig. 2) was unexpected and deserves further attention. Either way, future interrogation of these possibilities will be important.

Our results with MX2 fusions to leucine zippers imply the importance of protein dimerization for HIV-1 inhibition (Fig. 4). This conclusion corroborates recent mutagenesis studies performed in the context of full-length MX2, which indicate that dimerization, but not higher-order oligomerization, is necessary for antiviral function (14, 15). This feature of MX2 therefore appears to be another significant departure from the current models for MX1-mediated viral suppression (5, 10). Further work will be needed to understand the differing oligomerization requirements of the MX proteins for the inhibition of distinct viral families.
